# Challenges of Home Health Care During COVID-19 Outbreak in Iran

**DOI:** 10.30476/ijcbnm.2020.86511.1349

**Published:** 2020-10

**Authors:** Alireza Atashi, Ahmad Nejatian

**Affiliations:** 1 Department of E-Health, Virtual School, Tehran University of Medical Sciences, Tehran, Iran; 2 Department of Health Policy, Management and Economics, School of Public Health Tehran University of Medical Sciences, Tehran, Iran

Dear Editor

The pandemic of COVID-19 is the second one in the 21st century after H1N1 Swine Flu in 2009-2010, which infected more than 4.9 million and caused more than 327000 death as of 23 May 2020 worldwide. ^1^ This pandemic has overwhelmed the health systems, specially hospitals, and the need for ICU beds and ventilators increased while many of patients with mild symptom of COVID-19 could be recovered at home. ^2^

In addition, early discharge of hospitalized patient and referring for Home Health Care (HHC) would be a suitable strategy for decreasing the overload of patient in hospitals and its consequences such as nosocomial infections. On the other side, many patients who receive HHC for other conditions (usually old people with chronic disease) need special attention during the pandemic for ensuring continuation of care and prevention of COVID-19 threat. 

The first patient in Iran was officially reported on February 19, 2020 in Qom city. According to World Health Organization about 129 341 Iranians were infected by Novel Corona Virus and 7249 died till 23 May 2020.1 As the nurses are frontline health worker who provide services in hospitals as well as in home settings, Deputy for Nursing of MOHME published the guideline for Home care for COVID-19 patient and about 405 institute for Home Care services were active to care of patient in their homes. ^3^ Regardless of the availability of detailed information about hospitalized COVID-19 patients by means of integrated Hospital Information Systems (HIS), there is no information about the number, characteristics and health outcomes of people who receive professional HHC in Iran during this pandemic. It is not only the problem of Iran’s Health system, but European countries have also encounter with lack of home care data. ^4^

The history of HHC, as an industry in Iran, dates back to 1999 when the first bylaw for HHC was ratified by Health Minister. In recent years, Act on the Sixth Five-Year Economic, Cultural and Social Development Plan of Iran for 2017-2022 enforced the MOHME to expand the HHC in community and insurance coverage for HHC is on the High Council of Health Insurance’s agenda. Thus, nowadays home health care is becoming integrated in the health system of Iran more than ever.

One of the problems of HHC during the COVID-19 in Iran (at least in the beginning of epidemic) was lack of appropriate and adequate Personal Protective Equipment (PPE) for the staff who worked in HHC settings directly with patients and families and raised fear of the risk of cross-infections by the staff and patients. 

The lack of information and data about home care in Iran’s healthcare system, lack of adequate and accurate monitoring, and inability of supervising have been among the challenges of HHC in Iran, ^5^ which during COVID-19 epidemic became more challenging. The lack of data has weakened the ability of policymakers for evidence-based and timely decisions to combat the disease. 

Using Information Technology (IT) in HHC has many advantages such as gathering and sharing information, supervising and providing telenursing. The HHC teams and patients as well as policymakers can benefit from IT implementation in HHC. This issue is especially a priority in the disasters like COVID-19 pandemic when the health system needs integrated and real-time data. On the other hand, to minimize the contacts between nurses and patients aiming to decrease the cross-contamination, Telenursing can be used for frequent contact between the nurses and patients to manage the condition by telephone or video call without direct contact.

According to lessons learned from COVID-19 pandemic, using IT in Home Health Care, especially implementation of Home Health Care Information System and its integration into national Electronic Health Record (EHR) and using Telenursing are among the important factors for developing the role of HHC in the health system of Iran; this could help to better manage the outbreaks. It is not a simple policy to implement and needs a national determination and plan with multi-sectoral cooperation.
